# Taxonomy and biogeography of the Nearctic *Raphia* Hübner (Lepidoptera, Noctuidae, Raphiinae)

**DOI:** 10.3897/zookeys.421.7517

**Published:** 2014-06-27

**Authors:** B. Christian Schmidt, Gary G. Anweiler

**Affiliations:** 1 Canadian Food Inspection Agency, Canadian National Collection of Insects, Arachnids and Nematodes, K.W. Neatby Bldg., 960 Carling Ave., Ottawa, ON, Canada K1A 0C6; 2 E.H. Strickland Entomological Museum, 218 Earth Sciences Building, Department of Biological Sciences, University of Alberta, Edmonton, Alberta, Canada, T6G 2E9

**Keywords:** DNA barcode, *Populus*, incipient species, subspecies, parapatry

## Abstract

The taxonomic status and biogeography of the North American *Raphia* species is reviewed using adult morphology, larval host plants, geographic phenotypic variation, and variation of mtDNA COI barcode sequences. Lack of diagnostic morphological differences, combined with relatively low mtDNA barcode divergences and clinal phenotypic variation in key geographic regions indicate that the six previously recognized species of North American *Raphia* are best interpreted as parapatric subspecies. *Raphia frater abrupta* Grote, **stat. n.**, *R. f. coloradensis* Putnam-Cramer, **stat. r.**, *R. f. piazzi* Hill, **stat. n.**, and *R. f. elbea* Smith, **stat. n.**, are accordingly revised to subspecies of *R. frater* Grote. Type locality restrictions are provided for *Raphia abrupta* and *Raphia frater* and a neotype is designated for *Raphia frater* var. *coloradensis*.

## Introduction

*Raphia* Hübner is a small genus of the Holarctic region, with a single African species (Poole 1989) questionably congeneric. As the sole genus currently comprising the Raphiinae, the phylogenetic placement of *Raphia* has an interesting history. Most early works included the genus in older concepts of the Pantheinae. Smith (in Smith and Dyar 1898) excluded *Raphia* from the Pantheinae, but remained uncertain of its affinities within the Noctuidae. Hampson similarly excluded *Raphia* from the Pantheinae (Hampson 1913) and Acronictinae (Hampson 1909), and although never published, *Raphia* would presumably have been included in Hamspon’s volume covering “Ophiderinae” (Kitching 1984), a catch-all subfamily for noctuoids with fully quadrifine hindwing venation and lacking other specialized features emphasized by Hampson. Forbes (1954) and Franclemont and Todd (1983) maintained *Raphia* in Pantheinae, and it was not until recent times that Beck (1996) proposed a separate subfamily to accommodate *Raphia*. Two additional genera were recently thought to be related to *Raphia*: *Diloba* Boisduval (Fibiger et al. 2009) and *Aon* Neumoegen (Fibiger and Lafontaine 2005), the former resulting in a family-level synonymy of Raphiinae under Dilobinae (Fibiger et al. 2009). Molecular study and re-assessment of morphological traits has since shown that Raphiinae and Dilobinae are best retained as valid subfamilies (Zahiri et al. 2013). Similarly, *Aon* has subsequently been reclassified as belonging to Erebidae: Hypocalinae (Lafontaine and Schmidt 2010).

Three *Raphia* species occur in temperate Asia, one in southern Europe (Poole 1989), and until now six species were recognized in North America (Lafontaine and Schmidt 2010). The current species concepts of Nearctic *Raphia* are essentially unchanged from those proposed by Smith (1908), although *Raphia coloradensis* was revised to the synonymy of *Raphia frater* by Schmidt and Anweiler (2010) and *Raphia piazzi* (Hill 1927), described after Smith’s work. Hence, over a century has elapsed since Smith’s comprehensive synopsis of the Nearctic species. In this study, we revise the taxonomy of the six North American *Raphia* taxa based on geographic variation in adult phenotype, genitalia morphology, mtDNA barcode variation, and larval host plant use.

## Methods and materials

Adult genitalia were prepared following the methods of Lafontaine (2004). Cleaned, stained genitalia were stored and examined in 30% ethanol, and slide-mounted in Euparal before being photographed using a Nikon D200 digital camera. Distribution maps were generated using SimpleMappr (http://www.simplemappr.net/). Tree distribution maps were adapted from USGS (2013).

We examined approximately 4000 specimens during the course of this study, primarily those of the CNC, EME, MEM, USNM, and UASM. Specimen repository abbreviations are as follows:

AMNH American Museum of Natural History, New York;

ANSP Academy of Natural Sciences, Philadelphia, PA;

BIO Biodiversity Institute of Ontario, Guelph, Ontario;

BMNH The Natural History Museum (statutorily British Museum [Natural History]), London;

CNC Canadian National Collection of Insects, Arachnids and Nematodes, Ottawa;

CSU Colorado State University, Fort Collins;

EME Essig Museum of Entomology, University of California, Berkeley, California;

MEM Mississippi Entomological Museum, Mississippi State, MS;

UASM University of Alberta Strickland Museum, Edmonton, Alberta;

USNM National Museum of Natural History (formerly United States National Museum), Washington, D.C.

DNA extraction, PCR amplification, and sequencing of the COI barcode region were performed at the Canadian Centre for DNA Barcoding (CCDB) and followed standard protocols (Hebert et al. 2013; http://www.ccdb.ca/resources.php). Only sequence records greater than 500bp (range 500bp – 658bp) are included.

## Results and discussion

**Morphology.** Comparison of 20 genitalia dissections of each sex, representing all geographic entities, failed to reveal any diagnostic differences. The shape of the male valve apex and clasper varied slightly, but do so even within a single population. The shape of the inflated vesica, the most important diagnostic character in many noctuid species complexes, showed no discernible differences. Female genitalia were similarly conservative in variation. The European *Raphia hybris* Hübner, which is externally very similar to *Raphia frater* (and is in fact virtually indistinguishable from some *Raphia frater coloradensis* phenotypes), differs from *Raphia frater* in valve shape, vesica structure (including presence of spinules that are absent in *Raphia frater*) and shape and size of the corpus bursae. This indicates that *Raphia* genitalic morphology is not unusually homogeneous, where interspecific differences might be lacking.

**Figure 1.**
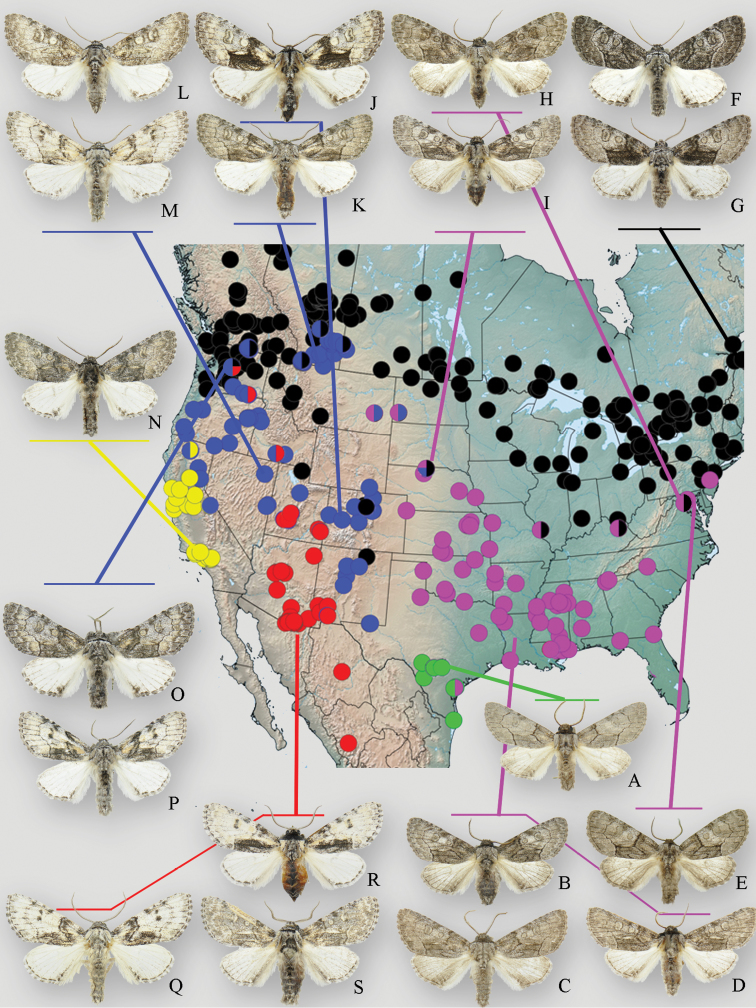
Geographic distribution and phenotypic variation of *Raphia frater* subspecies. Circles indicate specimens examined during this study: green – subsp. *piazzi*; pink – subsp. *abrupta*; black – subsp. *frater*; blue – subsp. *coloradensis*; yellow – subsp. *cinderella*. Multi-coloured circles indicate transitional populations and/or phenotypically intermediate specimens between respective subspecies. **a** *Raphia frater piazzi* (Zavallo Co., TX) **b** *Raphia frater abrupta* (Oktibeha Co., MS) **c** *Raphia frater abrupta* (Cottle Co., TX) **d** *Raphia frater abrupta* (Cottle Co., TX) **e** *Raphia frater abrupta* (Montgomery Co., MD) **f, g** *Raphia frater frater* (Edmunston, NB) **h** *Raphia frater abrupta* – *frater* intermediate (Anne Arundel Co., MD) **i** *Raphia frater abrupta* – *frater* – *coloradensis* intermediate from highly variable population in Cherry Co., NE **j** *Raphia frater coloradensis* (Alamosa Co., CO) **k** *Raphia frater coloradensis* (Milk River valley, AB) **l** *Raphia frater coloradensis* (Sanpete Co., UT) **m** *Raphia frater coloradensis* (Elko Co., NV) **n** *Raphia frater cinderella* (Ventura Co., CA) **o, p** *Raphia frater coloradensis* – *frater* intermediates (Chelan Co., WA) **q** *Raphia frater elbea* (Cochise Co., AZ) **r** *Raphia frater elbea* (San Juan Co., UT) **s** *Raphia frater elbea* (Santa Cruz Co., AZ). All specimens are males.

The North American *Raphia* species have previously been delineated based on wing colour and pattern (Smith 1908), and geographic variation is considerable (Fig. 1). The most important forewing characters include ground colour, extent of medio-anal black shading, shape of the antemedial band, and the amount of fuscous shading of the hindwing. The colour of the prothoracic setae and overall size also vary.

*Raphia* has an extensive North American distribution, occupying virtually all biomes. Phenotypes are generally quite consistent regionally, but can appear drastically different in geographically disparate areas, which led early authors such as Smith (1908) to recognize multiple North American species. To assess phenotypic and mtDNA variation in these contact zones, we therefore attempted to locate and study specimens from key geographic regions where either two or more taxa would be expected to occur sympatrically or transition from one phenotype to another. The most comprehensive data were available for four such regions: **a**) the central Great Plains and **b**) the northeastern U.S., both where nominal ssp. *frater* interacts with ssp. *abrupta*; **b**) southern New Mexico where ssp. *elbea* meets ssp. *coloradensis*, and **c**) the Pacific Northwest / northern Rocky Mountains where sspp. *coloradensis*, *frater* and *elbea* meet (Figs 1, 3).

In the central Great Plains, a large series of over 60 specimens from northern Nebraska (Cherry Co.) is so variable that scarcely two individuals are alike, varying from the granular, dark grey forewing and white hindwing of ssp. *frater*, to the even, light grey forewing and slightly fuscous hindwing of *Raphia frater abrupta*; an intermediate specimen is shown in Fig. 1i. Some individuals show the blotchy black and grey pattern (with a contrasting black medio-anal shade) characteristic of *Raphia frater coloradensis*. A shorter series from Kansas (Riley Co.) falls within the variation of the Nebraska population. Interestingly, Smith (1908) remarked that Denver, Colorado specimens varied more towards ssp. *abrupta* than ssp. *coloradensis*. Single specimens from southeastern Montana and southwestern North Dakota have relatively pale forewings, like ssp. *coloradensis*, but some have fuscous hindwing shading and a dark prothoracic collar like *abrupta*. The zone of clinal variation may therefore be more extensive than the specimens from the few available sites in the northern Great Plains indicate, so further surveying in the region from southeastern Montana and southwestern North Dakota southward to western Nebraska / Kansas and eastern Colorado (particularly along major river corridors) would be helpful. Such a large transition zone appears to be the result of continuous, flat topography with a single, widespread *Populus* L. species (*Populus deltoides* Bartr.) that is utilized by both ssp. *coloradensis* (to the northwest) and ssp. *abrupta* (to the southeast). This transition zone corresponds closely with the suture zone first proposed by Remington (1968) and recently verified by others (Swenson 2010 and references therein).

**Figure 2.**
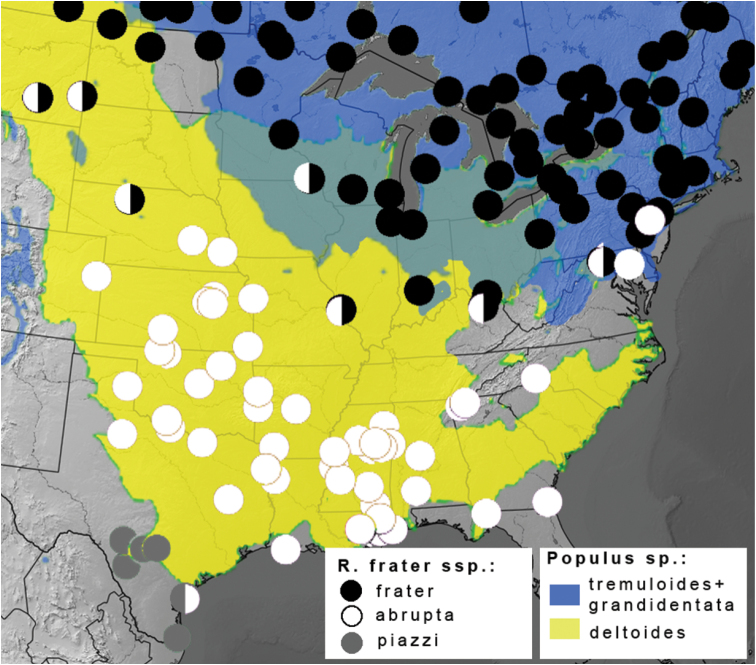
Distribution of *Raphia frater* subspecies (circles) relative to range of *Populus* larval host plants (coloured shading) in eastern North America. Black circles - subsp. *frater*; white circles – subsp. *abrupta*; grey circles – subsp. *piazzi*; half-circles represent transitional populations and/or phenotypically intermediate specimens. Blue shading – combined ranges of *Populus tremuloides* and *Populus grandidentata*; yellow shading – *Populus deltoides*; range overlap depicted in green. *Populus* ranges adapted from USGS (2013).

The nature of the ssp. *abrupta* – *frater* interface is somewhat different in the Northeast, and is seemingly more influenced by topography and host plant distribution (Fig. 2); at least three *Populus* species occur regionally among topography ranging from coastal floodplains to the Appalachian Mountains. Specimens from the Pocono Mtns. of Pennsylvania are *Raphia frater frater*, whereas nearby central Maryland (Ann Arundel Co.) specimens (Fig. 1h) show transitional features in having a forewing pattern much like ssp. *frater*, but with a fuscous hindwing and a darker prothoracic collar characteristic of ssp. *abrupta*. Coastal Maryland (Montgomery Co.) specimens are typical *Raphia frater abrupta* (Fig. 1e). As discussed below, the transition zone between ssp. *frater* and ssp. *abrupta* seems to be mediated by habitat and host plant differences, with *frater* largely associated with aspen (*Populus tremuloides* Michx. and *Populus grandidentata* Michx.; Fig. 2) and ssp. *abrupta* with cottonwood (*Populus deltoides*; Fig. 2). Study of the populations on either side of the Ohio River is needed because *frater* occurs throughout Ohio (Rings et al. 1992), whereas the few northeastern Kentucky specimens that were examined are mostly like ssp. *abrupta*, but show some ssp. *frater* traits, including a mostly white hindwing. The Ohio River valley is an important suture zone between other biota, but the relative limits of *Raphia frater frater* and ssp. *abrupta* from the northern Appalachians eastward appear to be further south than recognized suture zones (Swenson 2010).

**Figure 3.**
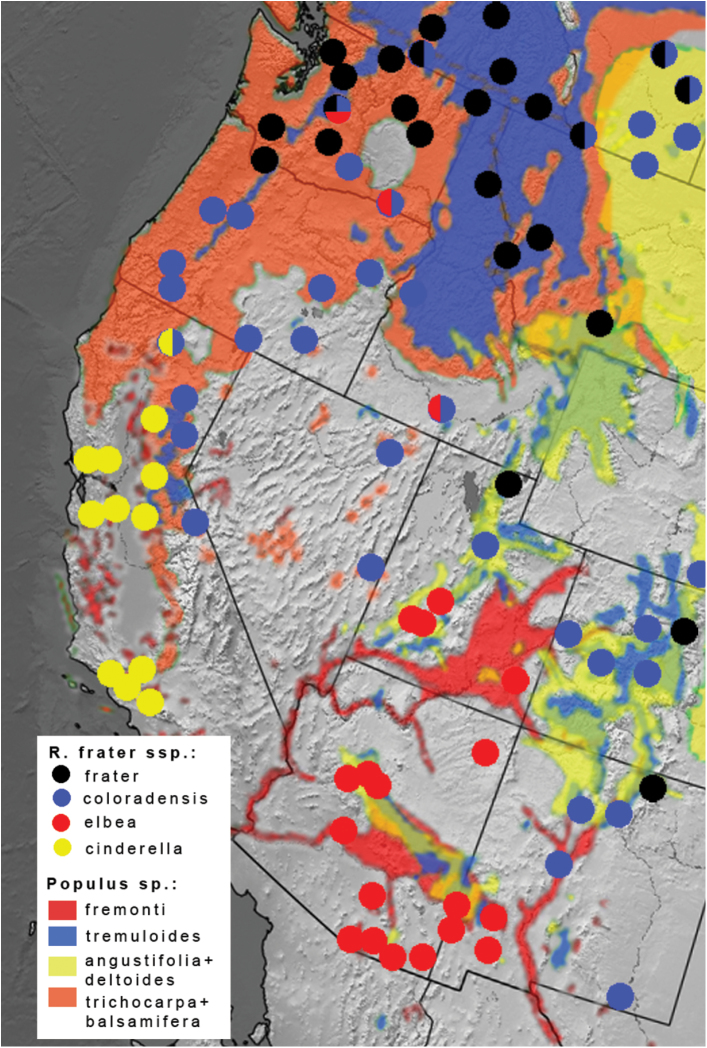
Distribution of *Raphia frater* subspecies (circles) relative to range of *Populus* species (coloured shading) in western North America. Half-circles represent transitional populations and/or phenotypically intermediate specimens. Ranges for *Populus trichocarpa* + *Populus balsamifera* and *Populus angustifolia* + *Populus deltoides* are combined, with both *Populus balsamifera* and *Populus deltoides* occuring in Alberta – Montana (upper right). *Populus* ranges adapted from USGS (2013).

In southern New Mexico where *elbea* and *coloradensis* meet, *elbea* is known from the Mimbres Mountains (Grant Co.) in the southwest, with the nearest documented *coloradensis* locations 180 km to the northeast in the Rio Grande valley, and 300 km to the east in Eddy County (Fig. 1). The phenotypic transition between the two taxa is more abrupt in southern New Mexico than it is in the Great Basin where *elbea* imperceptibly transitions to the very pale Great Basin forms of *coloradensis*. A series from Twin Falls, Idaho, and some individuals from Leavenworth, Washington (Fig. 1p), are indistinguishable from Arizona *elbea* (Fig. 1q). *Raphia frater elbea* therefore grades into *Raphia frater coloradensis* in low-elevation habitats of the northern Great Basin. Specimens from the Eddy Co., New Mexico population are most like *coloradensis*, but some individuals are again indistinguishable from *elbea*. The mtDNA haplotypes associate this population with *coloradensis* (Fig. 3), and the available larval hosts are *Populus angustifolia* James and *Populus deltoides* (National Park Service 2014), but not *Populus fremonti* Wats. with which *elbea* is most often associated. Analysis of *Raphia* populations from the lower Rio Grande valley of New Mexico is desirable given the geographically intermediate position between *elbea* and *coloradensis*, and the presence of *Populus fremonti* (Fig. 3).

Populations at the edges of the Great Basin can be extremely variable, much like the situation between *abrupta* and *frater* in the north-central Great Plains. Series of specimens from Waterton Lakes, Alberta; Okanagan Valley, British Columbia; Baker County, Oregon; and Leavenworth, Washington range from the typical dark grey *frater* to pale yellowish *coloradensis* (Fig. 1). The Leavenworth population is remarkable in that it exhibits phenotypes ranging from *frater* to *coloradensis* (Fig. 1o) and *elbea* (Fig. 1p).

The geographic structure of California populations is not well documented; typical *cinderella* occurs from the San Francisco Bay area southward through the Central Valley to Los Angeles Co., but *Raphia frater* is apparently absent from southeastern California and the southern Sierra Nevada. Northern California (including the Sierra Nevada) specimens are most like Great Basin *coloradensis* but the transition from *cinderella* to this paler form is subtle, with Siskiyou Mountains material appearing intermediate. The Siskiyou Mountains are part of a northern California – southern Oregon suture zone also identified for other flora and fauna (see Swenson 2010 and references therein). Areas in central Texas where *abrupta* and *piazzi* meet, and eastern New Mexico/west Texas where the ranges of *abrupta*, *coloradensis* and *elbea* converge, remain unstudied.

Extreme phenotypic variation is therefore the modal geographic pattern at suture zones. In all cases where we examined suture zones between putative taxa, phenotype variation was moderate to extreme, and specimens could not consistently be assigned to existing taxonomic categories. Similarly, mtDNA variation patterns show no evidence of sympatric, reproductively isolated taxa, as discussed below.

**Host plants.** 72% of the 132 larval collections of *Raphia frater* from across Canada summarized by Prentice (1962) came from trembling aspen, 17% from other *Populus* species (*Populus balsamifera* L., *Populus trichocarpa* Torr. & Gray, *Populus grandidentata*, *Populus* ‘× *canadensis*’ (Alt.), *Populus nigra* L. var. *italica* du Roi), and 3% from *Salix* spp. Three records from white birch and one from alder (both Betulaceae) reported by Prentice are exceptional and possibly accidental. Wagner et al. (2011) consider *Populus* to be the main hosts, and question the validity of records from birch and alder. The closely-related European species, *Raphia hybris*, is restricted to *Populus*. *Raphia* larvae possess an unusually large number of proleg crochets (Beck 1996), a trait also seen in the *Populus*-feeding genus *Ufeus* Grote (Noctuidae: Noctuinae); this trait is postulated to be an adaptation to maintaining a grip on the leaves of aspen species (Lafontaine and Walsh 2013), which tremble even in slight breezes. Based on these data, *Populus*, and to a lesser extent *Salix* L. (both Salicaceae), are certainly the primary and probably the only larval host plants. *Salix* may be used only incidentally or in certain regions/habitats; the parallel paucity of *Raphia* and *Populus* occurrence in the central Appalachian region is notable (Fig. 2). Eight species of *Populus* occur in North America, divided into four sections: *Leucoides* Spach (*Populus heterophylla* L.), *Aigeiros* Duby (*Populus deltoides*, *Populus fremonti*), *Tacamahaca* Spach (*Populus balsamifera*, *Populus trichocarpa*, *Populus angustifolia*) and *Populus* L. (*Populus grandidentata*, *Populus tremuloides*). One additional species, *Populus mexicana* Wesmael of central Mexico, is the sole constituent of section *Abaso* Eckenwalder (Eckenwalder 1996).

Although it is reasonably certain that *Raphia frater* larvae are Salicaceae specialists, the geographic variation in host use and extent of specialization is not well understood. Nonetheless, it is possible to extrapolate broader host use patterns based on larval collections, host plant distributions and habitat associations. Below, we outline some potential scenarios of host use among *Raphia frater* subspecies.

Host plant records for *Raphia frater frater* (Prentice 1962) indicate that *Populus tremuloides* is probably the dominant, and certainly the most geographically widespread host (with the caveat that the high proportion of trembling aspen collections may simply reflect sampling bias). In eastern North America, the southern range limit of *Raphia frater frater* corresponds closely with the combined southern limits of the two aspen species (section *Populus*: *Populus tremuloides* and *Populus grandidentata*; Fig. 2). *Raphia frater* populations in riparian habitats of southern Alberta, where *Populus tremuloides* is scarce or absent, are associated with other *Populus* species that form a complex zone of hybridization and overlap among four species (*Populus deltoides*, *Populus balsamifera*, *Populus trichocarpa*, and *Populus angustifolia*) along the major river valleys (Brayshaw 1965, Floate 2004). In this region *Raphia frater frater* phenotypes transition to *Raphia frater coloradensis* (Fig. 3). The northernmost extent of *Populus deltoides* and *Populus deltoides* × *balsamifera* bybrids in the Red Deer River valley at about 52° latitude (Floate 2004) also coincides with the northernmost extent of *Raphia frater coloradensis*-like phenotypes; north of there where *Populus tremuloides* is the dominant species of the Aspen Parkland ecoregion, only pure *Raphia frater frater* phenotypes occur. Similarly, transitional *frater-coloradensis* populations occur in southwestern Alberta, southern British Columbia and central Washington at the range edges of *Populus tremuloides*, where *Populus trichocarpa* becomes the dominant *Populus* (Fig. 3). In northern Labrador, *Raphia frater frater* is at its northeastern range limit (not shown), occurring beyond the range of *Populus*; *Salix* species are the presumed hosts.

Throughout most of the range of *Raphia frater abrupta*, *Populus deltoides* is the only *Populus* species present. Swamp cottonwood (*Populus heterophylla*) has a small eastern North American range, occurring primarily along the Mississippi and Ohio River valleys and along the Atlantic seaboard (see e.g., Sibley 2009, USGS 2013), so this may serve as a host in some areas. The hosts for the southwest Texas taxon *Raphia frater piazzi* are unknown, and may constitute willows rather than *Populus*, the latter being rare or absent where *Raphia frater piazzi* occurs (Fig. 3).

In the Pacific Northwest, *Raphia frater frater* is associated with *Populus tremuloides* in northern Washington and British Columbia, with *Raphia frater coloradensis* of dry, low-elevation habitats associated with *Populus trichocarpa* (L. Crabo, pers. comm.). Crumb (1956) documented a larval collection from the latter species in south-central Washington. Throughout most of the Pacific Northwest, the only *Populus* species are *Populus tremuloides* at upper elevations, and *Populus trichocarpa* in low-elevation riparian habitats and drier soils in moist regions (Fig. 3). A third species, *Populus angustifolia*, occurs locally in the eastern parts of the Pacific Northwest, with habitats similar to *Populus trichocarpa*. There are no specific host records for the Californian *Raphia frater cinderella*, with both *Populus trichocarpa* and *Populus fremonti* being the most likely hosts. Along the east slopes of the Oregon Cascade Ranges to the north, *Raphia frater coloradensis* associates with *Populus trichocarpa* (L. Crabo, pers. comm.) Dyar (1894) cites “poplar” as a foodplant for larvae from Yosemite. Subspecies *coloradensis* in the Sierra Nevada, to the east of the central Californian range of *cinderella*, is associated with *Populus tremuloides*.

Arizona populations of *Raphia frater elbea* feed on *Populus fremonti* (Crumb 1956; D. Wagner, pers. comm.), and again this is the only available *Populus* in much of the range of ssp. *elbea*, excepting the higher elevations in the mountain ranges of central Arizona where *Populus tremuloides* and *Populus angustifolia* occur (Fig. 3). A population of *Raphia frater coloradensis* in the Rio Grande valley of central New Mexico is also associated with riparian *Populus fremonti* (Fig. 3). Aspen-associated, *Raphia frater frater*-like populations may occur at high elevations in Arizona, similar to the situation in Colorado, but this has not been documented. This raises the interesting possibility that *frater* occurs at higher elevations, with *elbea* occurring in the low-elevation floodplain. Surveying at the uppermost elevations of the Chiricahua and Santa Catalina Mountains of southeastern Arizona where aspen occurs have so far not yielded *Raphia* (BCS unpubl. data), but the more expansive range of *Populus tremuloides* along, for example, the Mogollon Rim is poorly surveyed.

In summary, larval host plant associations of *Raphia frater* populations shows some broad congruencies between subspecies and *Populus* species distributions, but with limited evidence for high host fidelity: range edges of *Raphia frater* subspecies generally do not closely follow those of the various *Populus* hosts, suggesting that *Populus* availability rather than high host fidelity may be the limiting factor to *Raphia* distribution, and that climatic and topographic effects have a greater selective influence that does host plant specialization. To what extent these congruencies reflect common co-evolutionary trajectories, and what factors drive intraspecific divergence, would be a fascinating and fruitful area of study.

**Figure 4.**
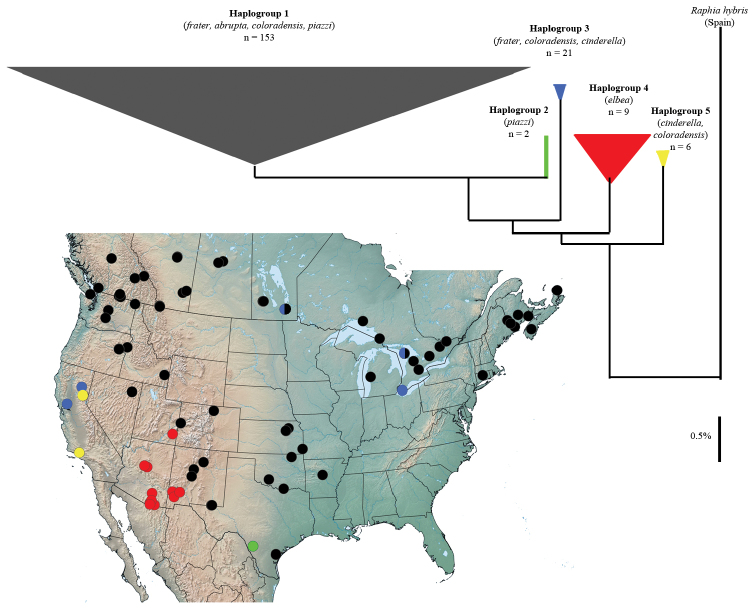
Neighbour-joining tree and associated sampling sites of mtDNA barcode haplotypes in *Raphia frater*. Haplogroup colour corresponds to that of sampling sites. Subspecies assignment based on morphology and sample size is indicated for each haplogroup. Width of triangles is proportional to number of haplotypes, height represents maximum divergence within haplogroup.

**Molecular variation.** The deepest splits in mtDNA barcode variation (alluded to previously in Lafontaine and Schmidt 2010) segregate North American *Raphia* into four groups, but only one of these is private to a recognizable taxon (*Raphia frater elbea*). None of the remaining subspecies exhibited discrete haplotypic variation. Based on analysis of 192 specimens from localities across the range of *Raphia frater* representing all subspecies (Suppl. material 1), haplotypes segregated into five groups: **1**) a large group from across most of the eastern, northern and central range portions that includes *Raphia frater frater*, *Raphia frater coloradensis*, *Raphia frater abrupta* and *Raphia frater piazzi*, varying by up to ~1.3% (Fig. 4); **2**) a discrete group closest to haplogroup 1, consisting of two *Raphia frater piazzi* specimens (Fig. 4); **3**) a divergent group of geographically disparate samples of Eastern *Raphia frater frater* (Ontario, Manitoba) and Californian *Raphia frater cinderella* specimens, differing by a minimum of ~2.2% from all other groups (Fig. 4); **4**) a group private to *Raphia frater elbea* (Arizona, New Mexico, Utah) with a minimum ~1.0% divergence; **5**) A group of Californian *Raphia frater cinderella* with a minimum divergence of ~1.8% from all other haplotypes.

The combination of California and eastern Canada samples in haplogroup 3 to the exclusion of all others was quite unexpected, given the geographic structuring of other haplogroups. Two haplogroup-3 populations (Bird Hill, Manitoba; Bruce Peninsula, Ontario) also exhibited group 1 haplotypes (Fig. 4), the only sampled populations to yield more than one haplogroup. Representative specimens from these sites were of the same phenotype and from the same sampling event. This haplogroup could therefore be a retained ancestral mtDNA polymorphism, or indicative of *Wolbachia*-induced mtDNA lineage sorting similar to that documented by Kodandaramaiah et al. (2013). Determining the underlying cause of this interesting variation will require study using nuclear gene markers and *Wolbachia* assays.

Considering the general lack of taxonomic resolution of North American *Raphia* in the barcode sequence, and comparing divergences among Palaearctic *Raphia* as a metric of mean species divergences within the genus, mtDNA variation is most parsimonious with a geographically structured, single-species interpretation. The contrast between often considerably different adult phenotypes and lack of significant mtDNA and morphological differentiation may reflect strong regional selection on bark-cryptic wing patterns, which in turn is dependent on dominant host trees that vary according to regional host preferences.

## Systematics

### Raphia

Taxon classificationAnimaliaLepidopteraNoctuidae

Hübner, [1821]

Rhaphia Agassiz, 1847. An unjustified emendation of *Raphia* Hübner, [1821].
Anodonta Rambur, 1858. Type species: *Noctua hybris* Hübner, [1813], by monotypy. A junior homonym of *Anodonta* Lamarck, 1799 [Mollusca].
Certila Walker, 1865. Type species: *Certila flexuosa* Walker, by monotypy. *Certila flexuosa* is a junior subjective synonym of *Raphia frater* Grote.
Saligena Walker, 1865. Type species: *Saligena personata* Walker, 1865, by monotypy. *Saligena personata* is a junior subjective synonym of *Raphia frater* Grote.

#### Type species.

*Noctua hybris* Hübner, [1813] by subsequent designation by Grote (1874).

### Raphia frater

Taxon classificationAnimaliaLepidopteraNoctuidae

Grote

#### Diagnosis.

Despite variation in adult facies and lack of a particular diagnostic trait, *Raphia frater* is recognizable by the combination of a broad, rounded forewing, often conspicuous antemedial and postmedial band, obsolete medial band (rarely faint), an orbicular, reniform and usually also a claviform stigma that are clearly outlined, black shading in the anal angle of the hindwing, and the conspicuously bipectinate male antennae. *Pseudopanthea palata* (Grote) and *Colocasia* Ochsenheimer species share some superficial similarities with *Raphia frater*, but attention to the above-stated characters relative to those in *Pseudopanthea* McDunnough and *Colocasia* Ochsenheimer will provide an easy diagnosis of this unique species.

#### Description.

**Figure 5.**
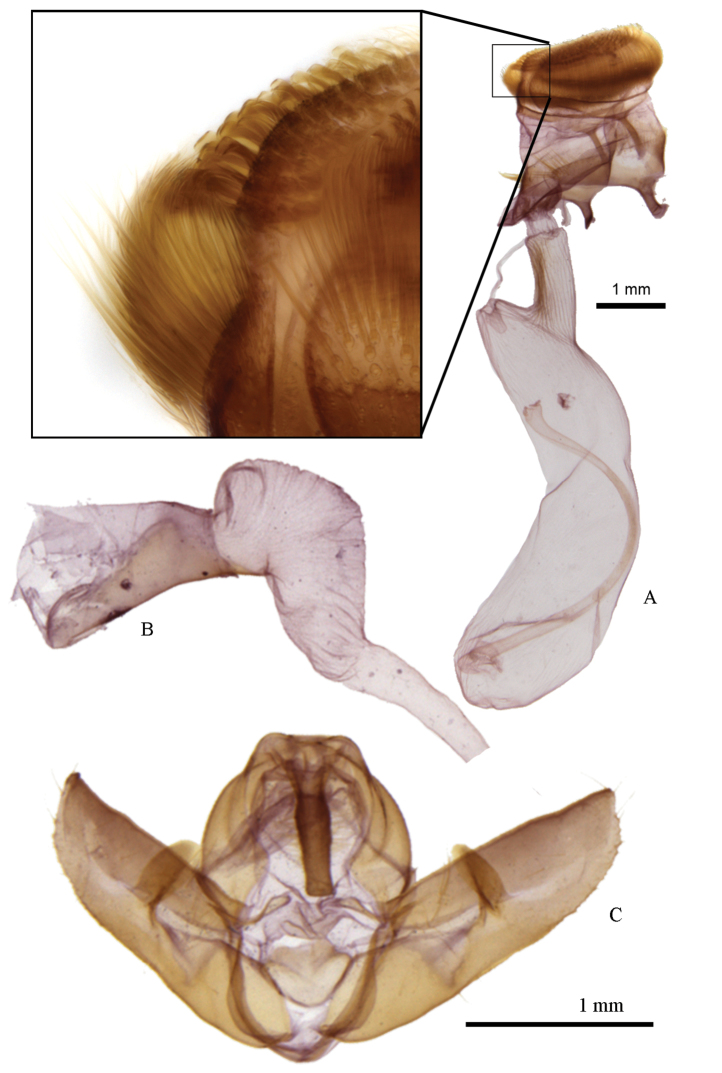
Genitalia of *Raphia frater*. **a** female (Konza Prairie Biol. Stn., Riley Co., KS; leg. Metlevski), with inset showing apical spatulate setae and subapical dense setal crown of papillae anales **b** male vesica **c** male genital capsule (Pitchfork Ranch, Grant Co., NM; leg. C. Ferris); note magnification difference between sexes.

**Head** – Male antennae bipectinate, anterior rami 3× longer than segment length, posterior rami 3.3× longer; female antennae simple; eyes round, with short, sparse interfacetal setae, visible only at high magnification; labial palpus with second segment clothed in long strap-like scales ventrally; third segment 0.6 × length of second segment (when denuded) and smoothly scaled; occiput and frons with mix of grey and black scales, frons with transverse line of black scales; frons rounded and moderately protuberant when denuded. **Thorax** – vestiture dark grey to yellowish grey, thoracic collar sometimes contrastingly darker than dorsum; tarsi smoothly scaled, with transverse bands of black and light to dark grey; tibia with similar scaling but with faint or indistinct banding; femur with long, shaggy hair-like scales. **Abdomen** – lacking specialized secondary sexual structures such as coremata; vestiture of smooth, short grey scales; small, rounded dorsal tufts on segments A3, A4 and A5, consisting of densely set spatulate scales. **Forewing** – ground colour varies from a dark charcoal grey to pale yellowish ochre; antemedial band a parallel-sided, double black line, varying from slightly irregular and rounded to nearly linear, acute, and angled at the cubital vein; medial band obsolete, usually reduced to a black bar or two diffuse lines at costa adjacent to reniform stigma, but band sometimes visible as a faint, diffuse black line extending from bottom of reniform stigma to anal margin; postmedial band a single black line, sinuate and slightly sagittate at veins (often faint or absent in ssp. *coloradensis* and *elbea*), expanding to diffuse black patch at costa; orbicular stigma paler than ground colour, with black border and often with a diffuse dark pupil (orbicular often absent entirely in ssp. *coloradensis* and *elbea*); reniform stigma paler than ground colour, with a black border (border often lacking in *coloradensis* and *elbea*) and a diffuse black central crescent; subterminal band absent, faint, or diffusely sagittate with paler distal edging; terminal area often darker grey than subterminal area. Average size is greatest in subspecies *frater*, while *abrupta* and *piazzi* are smallest; forewing length varies from 16.2 mm and 18.5 mm in male and female *Raphia frater frater* to 13.7 mm and 15.2 mm in male and female *Raphia frater abrupta*, respectively. **Hindwing** – ground colour varying from white, white and dusted with fuscous grey (ssp. *frater*, *coloradensis*, *elbea*, *cinderella*, *piazzi*), or entirely pale fuscous grey (ssp. *abrupta*), females with more fuscous than males; crescentic discal spot diffuse or absent; postmedial band faint or absent, although nearly always with a contiguous diffuse black patch at anal angle. **Male genitalia** (Fig. 5) – uncus slightly compressed dorsoventrally, with slight medial bulge, apex blunt; valva tapering more or less evenly to a rounded point, sacculus poorly differentiated from remaining valva; ampulla long and flattened, 0.7 × length of valva width, projecting mesially; aedeagus stout and sausage shaped, 2.1 × longer than wide; vesica a simple kidney-shaped, unarmed chamber equal in length to aedeagus, tapering gradually into ductus. **Female genitalia** (Fig. 5) – bursa copulatrix membranous, lacking apparent differential sclerotization, including ostium, ante- and postvaginal plate; ductus bursae a simple rugose tube, 3.3 × longer than diameter, connecting subbasally to corpus bursae; corpus bursae a simple kidney-shaped chamber, slighter wider over apical two-thirds; ductus seminalis arising from basal end of corpus bursae, caudad of ductus bursae; papillae anales short, bluntly rounded, with a broadly joined base, with two types of unusual, modified setae: 1) a dense band of thin, evenly curved setae arising from base of papillae and curving up to caudal margin, outer surface of lobe virtually encircled by a dense setal crown; and 2) highly modified thick, spatulate setae densely set along caudal margin of lobe. **Immature stages** – larva described by Thaxter (1883) based on eastern specimens, and by Dyar (1894) from Yosemite, California (possibly referable to *Raphia frater cinderella*). Illustrations in McCabe (1991) (head capsule and mandible), Wagner (2005) and Wagner et al. (2011). Mature larva stout, tapered only slightly anteriorly, bluish green to apple green with a slightly translucent quality, pinacula yellow, a dorsal transverse yellow band on A1, A5 and A8 extends to just above spiracle; T2 with short horn-like process middorsally, reddish with yellow base; these bands with whitish anterior border, those on A5 and A8 partially bordered with reddish orange; prolegs green, anal prolegs with yellow and reddish orange; head whitish green, usually retracted into T1, ocelli black, labrum whitish; total length 40 to 30 mm. Thaxter (1883) states that male larvae are more slender and smaller. Cocoon tough and firm, incorporating debris; pupa cylindrical with a rounded abdomen, cremaster short but broad and thick, lacking hooks. Eggs laid in small clusters or overlapping in linear groups; early instars much more elongate ‘semi-loopers’ with A3 and A4 prolegs reduced (Wagner et al. 2011). Larvae rest along midrib of leaf underside. Comparison among larvae of Californian *Raphia frater* (Dyar, 1894), *Raphia frater frater*, *Raphia frater abrupta* and *Raphia frater elbea* indicate no discernible differences among these subspecies.

**Biology and distribution.** *Raphia frater* occurs in virtually all wooded or shrubby habitats of the boreal region since the larval hosts form a dominant part of most non-coniferous forest types. It can be one of the most common late spring noctuids in aspen-dominated boreal forests of central Canada. In the West it becomes increasingly more restricted to riparian areas, particularly major river systems in drier regions of the Pacific Northwest and the desert of the Southwest. *Raphia frater* has a nearly transcontinental distribution, absent only from the arctic and most of the subarctic. The records from northern subarctic Labrador are surprising, but are based on three CNC specimens from two localities, so the data appear to be authentic. Handfield (2011) cites records from the northeast shore of the St. Lawrence, but the species is not known from Newfoundland. The range is essentially continuous south to northern Mexico, although very spotty throughout the Atlantic states, and spotty or absent in the central to southern Appalachians. Nominal *Raphia frater frater* occurs across the boreal region south to the northeastern States, southern Great Lakes region, and northern Rockies / Pacific Northwest; *Raphia frater abrupta* occurs from the Great Plains southward to eastern Texas and eastward to the Atlantic seaboard; *Raphia frater coloradensis* occurs from western California to the eastern slope of the Rockies; *Raphia frater elbea* occurs from at least southeastern Utah through Arizona, southwestern New Mexico and into Mexico; *Raphia frater cinderella* is restricted to western and central California; and *Raphia frater piazzi* occurs from the Edwards Plateau into southern Texas. *Raphia frater* is univoltine across the boreal region and most of the west, with peak flight activity from late May to July. It is bivoltine in the eastern U.S., flying mostly in April to May, and July to August. In the Deep South, *Raphia frater abrupta* has three abundance peaks: March, May and a smaller flight (partial third brood?) in September (Brou 2014). Larvae are most common from late July to mid-August in Canada (Prentice 1962).

### Raphia frater frater

Taxon classificationAnimaliaLepidopteraNoctuidae

Grote

Figs 1f , 1g , 2 , 3

Raphia frater Grote, 1864
Saligena personata Walker, 1865

#### Type material.

*Raphia frater* Grote, 1864 – # 7675 [ANSP]. Type locality: Middle States [eastern USA]; here restricted to Mount Pocono, Monroe Co., Pennsylvania. Grote (1864) simply stated the type locality as “Middle States,” and no additional information is available on the holotype label data. We interpret this as referring to the region south of the New England States, and north of the southern States. Given the complex variation of North American *Raphia frater*, it is advisable to restrict the type locality. As Grote’s material likely originated from the eastern United States, we restrict the Type locality to Mount Pocono, Monroe Co., Pennsylvania, from which we examined typical *Raphia frater frater* specimens. *Raphia frater* and *Raphia abrupta* are the oldest available names for this species, and were published simultaneously. As first revisers, we designate *frater* as the senior name (ICZN, Article 24.2.2). Syn. *Saligena personata* Walker, 1865 - [BMNH]. Type locality: United States.

#### Diagnosis and description.

The nominal subspecies of *Raphia frater* typically has an even, powdery, dark grey forewing ground colour with all of the markings complete, consisting of the antemedial and postmedial band, and the orbicular, reniform and usually the claviform stigmas. Average forewing length is 16.3 mm (*n* = 9) in males, 18.6 mm in females (*n* = 9). The male hindwing is white with little or no dusting of black scales in the subterminal area, and with a pronounced, diffusely-edged black patch in the anal angle, this often with an adjacent black line formed by the terminus of the postmedial band; females usually have some fuscous scales on the hindwing, especially on a slight postmedial band. This subspecies generally lacks the form with contrastingly darker medio-anal and costal black patches that is prevalent in *Raphia frater coloradensis*, but it does occur rarely even in Atlantic Canada (Fig. 1g). The yellowish-ochre forewing scales typical of *Raphia frater coloradensis* are absent. *Raphia frater abrupta* differs in having a more angulate and linear antemedial band, a paler grey and less powdery-appearing forewing, duskier hindwing, and smaller size. As discussed in the section on *Raphia frater*, geographically intermediate populations are extremely variable with respect to these traits, and are considered to be transitional between subspecies *frater* and *coloradensis*/*abrupta*, the only two subspecies abutting the range of *Raphia frater frater*.

#### Biology and distribution.

*Raphia frater frater* is primarily a boreal taxon, especially common in aspen (*Populus tremuloides* and *Populus grandidentata*) dominated forests and the Aspen Parkland ecoregion of the Prairie Provinces. In the East, it extends south of the Great Lakes region into Pennsylvania, Ohio and Indiana, but apparently not southward into the southern Appalachians, which are essentially devoid of *Raphia* records. The transition zone between *Raphia frater frater* and *Raphia frater abrupta* extends from Maryland westward roughly along the Ohio River Valley to east-central Missouri, then northwestward through the northern Great Plains. The southeastern range edge of *Raphia frater frater* is virtually identical to that of both trembling and bigtooth aspens (Fig. 2). In the West, *Raphia frater frater* occurs south along mid-elevation mountain ranges of the Pacific Northwest into Washington, and southward along the Rocky Mountains. Specimens from high elevations in Colorado (Gilpin Co., 9500’) and New Mexico (Sangre de Cristo Mtns., 7900’) are of the typical *frater* phenotype, the *coloradensis* phenotypes occurring at lower elevations.

### Raphia frater abrupta

Taxon classificationAnimaliaLepidopteraNoctuidae

Grote stat. n.

Figs 1b–e , 2

Raphia abrupta Grote, 1864
Certila flexuosa Walker, 1865

#### Type material.

*Raphia abrupta* - female holotype # 7675 [ANSP]. Type locality: not given; here restricted to Sycamore Landing, Seneca, Montgomery Co., Maryland. The female type bears no locality or collector label data, and since this is a widespread, geographically variable taxon, we restrict the type locality to Sycamore Landing, Seneca, Montgomery Co., Maryland; a series in USNM from this locality, collected by D. C. Ferguson, is phenotypically more similar to the female type than specimens from the Great Plains; it is also more likley that the holotype originated from the eastern US rather than the Great Plains, which were not well collected in the mid 1800’s.

*Certila flexuosa* Walker - [BMNH; not examined]. Type locality: North America.

#### Diagnosis and description.

*Raphia frater abrupta* replaces *Raphia frater frater* from the central Great Plains eastward to the mid-Atlantic seaboard, and southward to eastern Texas and Florida. It is on average smaller with a more evenly-coloured forewing, a more linear, angulate antemedial band and a fuscous hindwing. Average forewing length is 13.7 mm (*n* = 9) in males, 15.2 mm in females (*n* = 9). The thoracic collar is often darker than the dorsal thorax, not concolorous as in *Raphia frater frater*. The wing facies of subspecies *abrupta* is in many ways intermediate between *Raphia frater piazzi* of central and southern Texas and *Raphia frater frater* to the north, but the exact nature of the interface between *abrupta* and *piazzi* in Texas remains unstudied.

#### Biology and distribution.

Subspecies *abrupta* occurs south of the range of the aspen species favoured by *Raphia frater frater* larvae, and its riparian haunts suggest it feeds on eastern cottonwood (*Populus deltoides*), the only *Populus* species in much of its range. Swamp cottonwood (*Populus heterophylla*) and willows (*Salix* spp.) may also be suitable hosts. This subspecies is apparently rare on the Atlantic seaboard and absent altogether in the Appalachians. We examined only a single historical specimen from New Jersey (Trenton), with records north of there assignable to *Raphia frater frater*. All Ohio records were attributed to *Raphia frater frater* by Rings et al. (1992), although specimens with a pale grey forewing and dusky hindwing, traits of the *abrupta* phenotype, rarely occur as far north as southernmost Ontario (Toronto) and southeastern Minnesota (Fillmore Co.).

### Raphia frater piazzi

Taxon classificationAnimaliaLepidopteraNoctuidae

Hill

Figs 1a , 2

Paphia [sic] *piazzi* Hill, 1927.

#### Type material.

Holotype male [USNM]. Type locality: Brownsville, Texas [USA].

#### Diagnosis and description.

*Raphia frater piazzi* is the least-known member of the group with a restricted distribution in central and southern Texas. Most similar in size and facies to *Raphia frater abrupta*, it is distinguished from that subspecies by the paler, more evenly grey forewing with sharper transverse lines than in *Raphia frater abrupta*. The biology and biogeographic relationship to *Raphia frater abrupta*, which occurs to the northeast of *piazzi’s* range, is not known, and very few specimens of this taxon are present in collections. An additional enigma is whether or not Rio Grande *piazzi* populations interact with the vastly-different looking Sonoran *Raphia frater elbea*.

#### Biology and distribution.

Described from southernmost Texas, this subspecies is otherwise known only from the Edwards Plateau region; a single specimen from Sinton County to the southeast is phenotypically intermediate between *abrupta* and *piazzi*, but clearly more field work is needed to establish the limits of both subspecies. mtDNA barcode data of three *piazzi* specimens (Sinton Co. and Zavalla Co.) are very similar to the haplotypes of *Raphia frater abrupta*, *Raphia frater frater*, and *Raphia frater coloradensis*.

#### Remarks.

We were unable to obtain DNA sequence from topotypical specimens of *piazzi* from the lower Rio Grande near Brownsville, Texas. The unique haplotype of the Edwards Plateau specimens (Fig. 4) may represent nominal *piazzi*, but could equally represent a unique genetic lineage from the Edwards Plateau, with its unique fauna much of which is not shared with the Rio Grande fauna.

### Raphia frater coloradensis

Taxon classificationAnimaliaLepidopteraNoctuidae

Putnam-Cramer stat. r.

Figs 1j–m , 1o , 3

Raphia frater var. *coloradensis* Putnam-Cramer, 1886
Raphia pallula H. Edwards, 1886, **syn. nov.**

#### Type material.

*Raphia frater* var. *coloradensis* - Neotype female, here designated [CNC]. Type locality: Deer Creek Cyn. Park, 39°33.18'N, 105°08.49'W, 5950’, SW Littleton, Jefferson Co., Colorado. None of the original types, three males and four females “taken in Colorado by D. Bruce,” could be located and are presumed lost. The primary type of *Xylena thoracica* Putnam-Cramer, the only other noctuid named by Putnam-Cramer, is housed at USNM. Prior to 1886, D. Bruce collected in the mountains and foothills near Denver (Brown 1966), and we accordingly select a specimen from the same region to designate as **neotype:** “Colorado: Jefferson Co. / 39°33.18'N, 105°08.49'W / Deer Creek Cyn. Park / SW Littleton, w of hogback / 16–17 June 2008, 5950’ elev / riparian area s. of road / leg: Chuck Harp uv trap”; “Neotype / *Raphia frater* var. / *coloradensis* Putnam-Cramer / Schmidt and Anweiler 2014.”

*Raphia pallula* - Holotype female [AMNH]. Type locality: Siskiyou Co., California [USA]. Published several months after *coloradensis* Putnam-Cramer, Edwards was apparently not aware of Putnam-Cramer’s name as it is not mentioned in his description.

#### Diagnosis and description.

Within the range of *coloradensis*, specimens identical to the typical boreal *Raphia frater frater* are often present; in the most arid parts of the range of *coloradensis* in the southern Great Basin, *coloradensis* is more consistently pale ochre yellow with obsolete transverse lines and diffuse black costal/reniform blotches, overall very similar to *elbea*, but with less pronounced costal and reniform dark patches. Average forewing length is 14.9 mm (*n* = 9) in males, 16.8 mm in females (*n* = 6).

#### Biology and distribution.

This subspecies occurs from southernmost British Columbia / Alberta to New Mexico, Utah, and California. It is most commonly associated with riparian, low-elevation habitats. Northern populations fly from late May to July in a single generation. Flight dates spanning from May into August in the Great Basin and Southern Rocky mountain region indicate a second or partial second generation.

#### Remarks.

*Raphia frater coloradensis* is the most weakly-differentiated subspecies, and may simply be an ecologically induced phenotype of *Raphia frater frater* that occurs in the warmer, drier regions of the West. Several populations, spanning a large geographical area, have been identified that exhibit a large range of phenotypic variation, as discussed above in the ‘Morphology’ section. Specimens from Siskiyou Co., California and the east slope of the northern Sierra Nevada (Sierra Co.) are phenotypically very similar to Great Basin *coloradensis*, and we therefore treat *pallula* as a junior subjective synonym. DNA barcodes of two specimens from the northern Sierra Nevada (Sierra Co.) belonged to the *frater-coloradensis-abrupta* haplogroup (Fig. 4).

### Raphia frater cinderella

Taxon classificationAnimaliaLepidopteraNoctuidae

Smith stat. n.

Figs 1n , 3

Raphia cinderella Smith, 1903.

#### Type material.

A male lectotype was designated by Todd (1982) [AMNH]. Type locality: Los Angeles Co., Cal. [California, USA].

#### Diagnosis and description.

*Raphia frater cinderella* is a Californian subspecies that is similar in size and colour to *Raphia frater coloradensis*, but with a more diffuse, poorly contrasting forewing pattern that usually lacks the pronouncedly darker reniform and costal dark patches. The forewing ground colour is also pale powdery grey, not pale ochre as it often is in *coloradensis*. The two taxa appear to intergrade in the Siskiyous and northern Sierra Nevada.

#### Biology and distribution.

The range of this subspecies is restricted to central and southern California west of the Sierra Nevada. Fremont Cottonwood and willows are the most likely larval hosts, although records specific to this subspecies are lacking. Most collection dates are from June; Records from Stanislaus Co. for April - May and July may indicate a second generation.

### Raphia frater elbea

Taxon classificationAnimaliaLepidopteraNoctuidae

Smith stat. n.

Figs 1q–s , 3

Raphia elbea Smith, 1908

#### Type material.

A male lectotype was designated by Todd (1982) [AMNH]. Type locality: Deming, [Luna Co.,], New Mexico [USA].

#### Diagnosis and description.

*Raphia frater elbea* is most similar to the pale yellowish-ochre forms of *Raphia frater coloradensis*, but differ from that subspecies in having both the costal and reniform dark patches more prominent; when present, the black medio-anal patch is also darker and more elongate; additionally, *Raphia frater elbea* appears to exhibit a unique, divergent mtDNA haplotype group.

#### Biology and distribution.

This subspecies occurs from southeastern Utah and western New Mexico southward through Arizona into northern Mexico. In southeastern Arizona it occurs in riparian areas in association with the larval host, *Populus fremonti*. Flight records are from February to October, with most being from March to May and August to September, indicating at least two generations annually.

## Conclusions

The North American *Raphia* populations exhibit considerable geographic variation in phenotype, previously segregated into six species. Despite these geographically structured phenotypic differences, diagnostic morphological differences in genitalia and larvae are not evident. Scrutiny of geographic contact zones between putative taxa revealed populations with extensive phenotypic and conservative molecular variation, rather than bimodal phenotypic variation coupled with deep molecular divergences that would be expected for sympatric, reproductively isolated taxa. *Raphia frater* larvae are not highly restricted to a host species or genus, but do specialize on *Populus* and *Salix*, with a pattern of regional host availability and possibly also preference. Differences in host plant suitability among the various species of Salicaceae remain unstudied. Assessment of morphology, mtDNA variation, and biogeography therefore leads us to conclude that the geographic segregates of North American *Raphia* are best treated as subspecies of a single species. The regional adaptation to habitats representing nearly all North American biomes, combined with relatively discrete geographic ranges of unique adult phenotypes, suggest a pattern of young or incipient species in the *Raphia frater* group.

The taxonomy and biogeography of the North American *Raphia* populations is a complex interplay between topography, host plant use, phenotypic variation and evolutionary history. This study is only the first attempt at a better understanding of this interesting group. Many questions remain unanswered: what are the exact geospatial and host plant patterns of the contact zone between *Raphia frater abrupta* and *Raphia frater frater*? Is there geographic overlap with altitudinal segregation in the West between aspen-feeding *frater* and cottonwood feeding *elbea*? Does the mtDNA haplogroup 3 represent *Wolbachia* infection? Do the lower Rio Grande / Edwards Plateau *piazzi* populations grade into *abrupta*? *Raphia* would provide a fertile area of study in understanding large-scale patterns of host plant use and biogeography of a widely distributed continental Lepidopteran.

## Supplementary Material

XML Treatment for Raphia

XML Treatment for Raphia frater

XML Treatment for Raphia frater frater

XML Treatment for Raphia frater abrupta

XML Treatment for Raphia frater piazzi

XML Treatment for Raphia frater coloradensis

XML Treatment for Raphia frater cinderella

XML Treatment for Raphia frater elbea

## Supplementary material

Supplementary material 1 Table S1. Specimen data for mtDNA barcode vouchers. doi: 10.3897/zookeys.421.7517.app1 Data type: data spreadsheet Explanation note: Haplogroup numbers refer to those given in Fig. 4; Abbreviations for specimen depositories are as given in Methods and materials section. This dataset is made available under the Open Database License (http://opendatacommons.org/licenses/odbl/1.0/). The Open Database License (ODbL) is a license agreement intended to allow users to freely share, modify, and use this Dataset while maintaining this same freedom for others, provided that the original source and author(s) are credited.B. Christian Schmidt, Gary G. Anweiler
